# Examining the Effects of Cognitive Behavioral Therapy With a Virtual Agent on User Motivation and Improvement in Psychological Distress and Anxiety: Two-Session Experimental Study

**DOI:** 10.2196/55234

**Published:** 2024-10-15

**Authors:** Katja Frischholz, Hiroki Tanaka, Kazuhiro Shidara, Kazuyo Onishi, Satoshi Nakamura

**Affiliations:** 1 Department of Psychology University of Regensburg Regensburg Germany; 2 Department of Information Science Nara Institute of Science and Technology Ikoma Japan; 3 Division of Arts and Sciences International Christian University Mitaka Japan

**Keywords:** cognitive behavioral therapy, cognitive restructuring, motivation, virtual agent, automatic negative thoughts

## Abstract

**Background:**

Cognitive behavioral therapy (CBT) is a valuable treatment for mood disorders and anxiety. CBT methods, such as cognitive restructuring, are employed to change automatic negative thoughts to more realistic ones.

**Objective:**

This study extends on previous research conducted by the authors, focused on the process of correcting automatic negative thoughts to realistic ones and reducing distress and anxiety via CBT with a virtual agent. It was aimed to investigate whether the previously applied virtual agent would achieve changes in automatic negative thoughts when modifications to the previous experimental paradigm are applied and when user motivation is taken into consideration. Furthermore, the potential effects of existing participant knowledge concerning CBT or automatic thoughts were explored.

**Methods:**

A single-group, 2-session experiment was conducted using a within-group design. The study recruited 35 participants from May 15, 2023, to June 2, 2023, via Inter Group Corporation, with data collection following from June 5 to June 20, 2023, at Nara Institute of Science and Technology, Japan. There were 19 male and 16 female participants (age range: 18-50 years; mean 33.66, SD 10.77 years). Participants answered multiple questionnaires covering depressive symptomatology and other cognitive variables before and after a CBT session. CBT was carried out using a virtual agent, who participants conversed with using a CBT dialogue scenario on the topic of automatic negative thoughts. Session 2 of the experiment took place 1 week after session 1. Changes in distress and state anxiety were analyzed using a Wilcoxon signed-rank test and *t*-test for paired samples. The relationships of motivation with cognitive changes and distress or anxiety changes were investigated via correlation analysis. Multiple linear regression was used to analyze the potential predictive qualities of previous knowledge of CBT and automatic negative thoughts regarding outcome measures.

**Results:**

Significant reductions in distress (all *P*<.001) and state anxiety (all *P*<.003) emerged throughout the first and second experimental sessions. The CBT intervention increased participants’ recognition of their negative thinking and their intention to change it, namely their motivation to change it. However, no clear correlations of motivation with changes in distress or anxiety were found (all *P*>.04). Participants reported moderate subjective changes in their cognition, which were in part positively correlated with their motivation (all *P*<.007). Lastly, existing knowledge of CBT did not predict reductions in distress during the first session of the experiment (*P*=.02).

**Conclusions:**

CBT using a virtual agent and a CBT dialogue scenario was successful in reducing distress and anxiety when talking about automatic negative thoughts. The promotion of client motivation needs to be critically considered when designing interventions using CBT with a virtual agent, and further experimental investigations on the causal influences between motivation and outcome measures need to be conducted.

## Introduction

### Theoretical Background

With regard to the question of what a human being is made of, a particularly popular model is that of Beck et al [1] who proposed that humans experience thoughts and feelings and show behavior. These 3 components are highly interrelated and influence each other positively and sometimes negatively. While some of our thinking patterns are clearly apparent to us, others tend to be harder to perceive by oneself. One theoretical model of thinking focused on negatively distorted thinking patterns [1,2]. These are referred to as automatic negative thoughts. Critically, it is difficult to catch these negative distortions in thinking because, as the name suggests, they occur automatically and most of the time subconsciously [1].

For mood disorders, such as depression and anxiety, cognitive interventions have been shown to be effective in reducing the respective symptomatology [3,4]. Techniques, such as those involving cognitive restructuring applied in cognitive behavioral therapy (CBT), are used to elevate negative moods and modify negative cognitions to be more realistic [1,2]. This is done by identifying automatic negative thoughts, discussing their validity and whether they are realistic, and establishing alternative thinking patterns [5,6]. Critically, an awareness of automatic negative thoughts needs to be established first, as an active effort to modify these is necessary [1].

Considering ongoing mental health care staff shortages and struggles with the financial feasibility of psychotherapy interventions [7,8], a need for additional support in mental health care has emerged. One way to aid this need for support is the use of technology to support clients, which can provide them with the care they need while relieving the mental health care system. For example, by implementing CBT techniques in mental health apps (see [9] for a review) or delivering CBT via conversational agents in the form of robots [10] or virtual agents (see [11,12] for reviews), clients can receive mental health interventions with little to no help from a human provider. These previous studies indicated the possibility of easing depressive and anxious symptomatology by applying CBT via conversational agents [13,14]. These effective technology-supported interventions are more flexible regarding time and location of use than standard psychotherapy with a human practitioner. The previously mentioned studies showed that the use of conversational agents can be an effective way of delivering CBT and that such approaches could potentially be used as supportive measures in the struggling mental health care system.

One topic that has long been of great interest in psychology and psychotherapy research is that of motivation [15-18]. Crucially, it has been found to be an important factor in predicting psychotherapy outcomes, with higher motivation typically related to more positive outcomes [15,17,18]. However, research shows that clients sometimes lack motivation for treatment at the beginning of psychotherapy [16] and highlights the need to ensure motivation for psychotherapy and efforts to increase it, if necessary. From a technological point of view, motivation also seems to play a central role as higher motivation has been linked with higher acceptance of and willingness to use technology [19]. Moderate to high dropout rates have been reported for internet-based psychotherapy interventions (see [20] for a review). Therefore, the technological application of psychotherapy techniques encompasses an elevated need for ensuring high motivation of clients and consequently requires heightened attention. While in the application of conversational agents in mental health care, the use of motivating character types for an agent is seemingly common, the evaluation of the effects of the agent on user motivation is a lesser explored outcome measure and not routinely considered when testing conversational agents for mental health care [11,12].

Previous experiments conducted by the authors of this study have focused on the application of a virtual conversational agent for delivering CBT and especially cognitive restructuring as a technique applied in psychotherapy [21,22]. Specifically, the process of correcting automatic negative thoughts to more realistic ones and consequently reducing negative moods was the focus of this intervention [22]. In these previous studies, a scenario-based CBT session with a virtual agent was used in a single-session experiment aiming to lead participants to recognize existing automatic negative thoughts and find more realistic alternatives for them. Shidara et al [22] found that negative mood (distress) could be reduced significantly via CBT with this virtual agent. However, there were some limitations to these works of research that the authors aim to overcome in this study. First, a single-session design is not a realistic enough replication of real-life CBT, which, in Japan, usually consists of multiple sessions (up to 16 in total) [23]. Second, the motivation of participants was not considered in these studies. To extend the previous study of Shidara et al [22] and counter its limitations, this research was conducted using the same human-agent interaction technology applied in the previous study with changes applied to the CBT scenario script and experiment procedure as described below. With these modifications, this study aims to gain valuable insights into relevant factors influencing the application of the described psychotherapy techniques delivered via the virtual agent for detecting and counteracting automatic negative thoughts, which can consequently enhance their effectiveness as measures of support within the mental health care system.

### Hypotheses

The first goal of this study was to extend the previous experiment by Shidara et al [22] by an additional session to make for a more realistic representation of real-life CBT concerning automatic negative thoughts in an experimental setting. The second goal was to investigate the role of motivation in changing automatic negative thoughts using CBT with a virtual agent. Furthermore, as the authors expected participants’ existing knowledge, especially concerning automatic negative thoughts, to be low, the study aimed to explore any influences of this knowledge being present or not on the outcome measures. To achieve these goals, a multiple-session experiment was carried out with statistical analysis based on the following hypotheses:

- Hypothesis 1: Distress and anxiety of participants are positively influenced by interaction with the virtual agent and therefore decrease.
- Hypothesis 2: Motivation of participants increases throughout the experiment.
- Hypothesis 3: Changes in psychological distress and anxiety correlate positively with participants’ motivation to change their negative thinking.
- Hypothesis 4: Cognitive change correlates positively with participants’ motivation to change their negative thinking.
- Hypothesis 5: Participants who have prior knowledge of automatic negative thoughts or CBT show bigger changes in psychological distress, anxiety, and cognition.

## Methods

### Participants

Participants for this study were recruited from May 15, 2023, to June 2, 2023, via Inter Group Corporation. To be eligible for this study, all participants were required to state normal hearing and to be native Japanese speakers. No specific requirements for depression or anxiety levels were set for participant recruitment. People of any gender and age could participate in order for the sample to be as representative as possible for the general population. In total, 35 participants completed both sessions of the experiment from June 5 to June 20, 2023, at Nara Institute of Science and Technology, Japan. This sample size was deemed sufficient based on comparisons with past experiments on the topic (see [11,24]). Overall, 19 males and 16 females participated, with their ages ranging from 18 to 50 years (mean 33.66, SD 10.77). Of the 35 participants, 11 (31%) were university students, 5 (14%) reported being either housewives or on leave of absence, 1 (3%) did not state their occupation, and 18 (51%) reported being employed. All participants were asked if they knew about automatic negative thoughts or CBT before the experiment. Five participants stated prior knowledge of automatic negative thoughts and 13 stated prior knowledge of CBT. Furthermore, participants were screened for signs of depression using the Japanese version of the Quick Inventory of Depressive Symptomatology self-report version (QIDS-SR) [25,26], and the mean score was 6.66 (SD 4.47), which indicated mild depression of a level similar to that measured in a previous study by the authors [22]. Originally, one more person participated in session 1 of this experiment. However, this person did not return for session 2, and consequently, this person’s data have been excluded from the analysis.

### Ethical Considerations

Institutional ethics approval has been received from the Nara Institute of Science and Technology where the experiment was carried out (approval number: 2019-I-24). All participants were informed prior to the experiment that they would be communicating with a computer-generated character about their mental health and that their data would be anonymous, and then, they gave informed consent to participate in this study. It was possible for participants to withdraw from the study if they desired. Participants received monetary rewards of up to 16,000 Japanese yen (US$ 115.30), depending on factors such as their living address.

### Outcome Measures

#### Primary Outcome Measures

For the experiment, multiple questionnaires (Japanese versions) were used to investigate the research questions. Depending on licensing and availability, the questionnaires were either administered in paper form or using the online questionnaire website SoSci Survey [27], which was accessed during the experiment.

The State-Trait Anxiety Inventory (STAI [28], Japanese version [29]) was administered in its paper form to measure participants’ state and trait anxiety. As a measure of psychological distress, the Kessler Psychological Distress Scale in its 6-item format (K6 [30], Japanese version [31]) was used and embedded in the online questionnaire. At the beginning of session 1, K6 was administered using its original wording, while at the end of session 1, the beginning of session 2, and the end of session 2, the wording was modified to ask about distress in the current moment.

To assess motivation, the original version of the Stages of Change Readiness and Treatment Eagerness Scale (SOCRATES) questionnaire [32] was modified and translated to Japanese to be used in the experiment. The Japanese version of the questionnaire [33] was used as a reference for the translations. Originally, the SOCRATES asks about alcohol or drug consumption [32]. The wordings of the 19 items were adapted so that instead of asking about these topics, the questionnaire asked participants about their negative thinking. The modified English version is included in Multimedia Appendix 1. The Japanese version is available from the corresponding author upon reasonable request. This modified version of the SOCRATES was administered digitally at the beginning of session 1 and at the end of both sessions 1 and 2.

As CBT and cognitive restructuring are focused on changing cognitive patterns, the Cognitive Change Immediate scale (CCI) [34] (translated to Japanese) was administered digitally at the end of sessions 1 and 2 to measure the short-term cognitive effects of the intervention. The Cognitive Change Sustained scale (CCS) [34] (translated to Japanese) was administered digitally at the beginning of session 2 to measure lasting cognitive effects following the first session.

#### Other Measures

As in the preceding study carried out by Shidara et al [22], the 16-item QIDS-SR [25] (Japanese version [26]) was administered digitally at the beginning of session 1 to screen participants for signs of depressive symptoms. Separate from the main hypothesis, the World Health Organization Quality of Life BREF questionnaire (WHOQOL-BREF [35], Japanese version [36]) was administered in paper form as a measure of participants’ satisfaction with their current life, and the General Self-Efficacy Scale (GSES [37], Japanese version [38]) was administered as a measure of participants’ self-efficacy. The Ten-Item Personality Questionnaire (TIPI [39], Japanese version [40]) was administered via the online questionnaire in session 1 to check for any influences of personality on the experiment’s outcome.

Lastly, as the reception of the virtual agent employed in this experiment, its role as an interlocutor, and the potential to build a relationship with the participants were of great interest, the Working Alliance Inventory-Short Revised (WAI-SR [41], Japanese version [J-WAI-SR] [42]) was administered digitally at the end of sessions 1 and 2 in order to gain insights into the participants’ perception. Furthermore, the digital questionnaire used in session 1 contained 9 questions on demographic information. Both digital questionnaires of sessions 1 and 2 contained a free answer item for participants to include any relevant thoughts concerning the experiment.

### Technological Components

**Figure 1:**
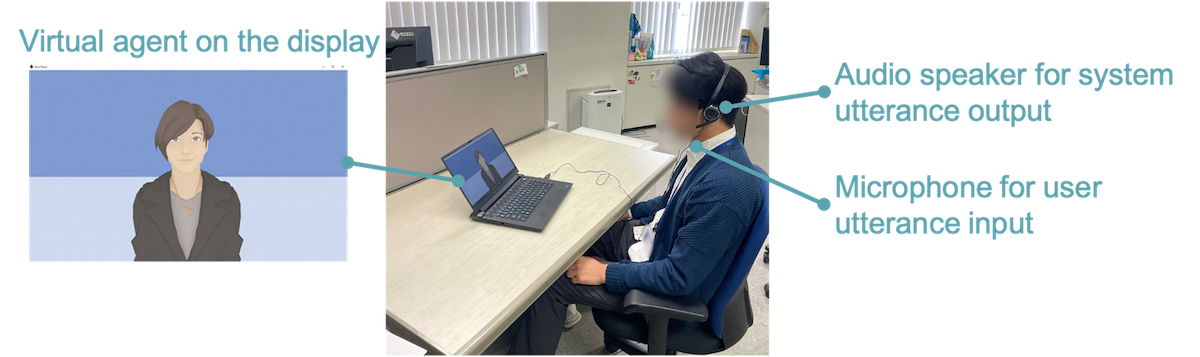
An insight into the human-agent interaction in the cognitive behavioral therapy experiment.

Concerning technological materials, a laptop, headset, and computer mouse were used by participants to fill in the digital questionnaires and interact with the virtual agent. Questionnaires and the virtual agent were displayed on the laptop screen. An image of the virtual agent used for the present experiment is provided in Figure 1. All questionnaires were answered via text input or by choosing adequate answer options. For the CBT session with the virtual agent, participants listened to the generated voice output through the headphones, and their verbal answers were picked up by the headset’s microphone. Simultaneously, their face was recorded on video. More details on the technology underlying the virtual agent used in the present experiment can be obtained from the previous work of Shidara et al [22] whose framework was applied here.

### CBT Dialogue Scenario

For the virtual agent to communicate with the participants, a CBT dialogue script was used. This script was taken from the previous study of Shidara et al [22] but modified to achieve a more natural and realistic sounding experience of psychotherapy or CBT, including phrases to validate participants’ utterances. This modification was carried out as empathetic responses have been linked to better user experience with conversational agents [11]. For this purpose, the wording and order of utterances were changed, and some utterances were added to the scenario. The translations of the modified Japanese scenarios for sessions 1 and 2 are included in Multimedia Appendix 2. The Japanese version is available from the corresponding author upon reasonable request.

### Procedure

#### Session 1

The general procedure of this experiment was based on the approach presented by Shidara et al [22]. During all steps of the experiment, participants could take as much time as needed. For the first experiment session, participants arrived at the laboratory and first received information on the experiment’s purpose and privacy. They then signed an informed consent form and were guided to sit in front of the laptop which would later be used. Next, the participants filled out 3 paper-form questionnaires: STAI, GSES, and WHOQOL-BREF. After that, they continued to fill in the digital questionnaire for session 1. After filling in the first modified SOCRATES, the digital questionnaire asked the participants to stop and call for the experimenter who then provided them with a digital leaflet containing information on CBT, a document containing explanations of automatic negative thoughts, and a paper with written explanations on how to interact with the virtual agent. After reading all explanations and if no more questions remained, the experimenter instructed the participants to put on the headset. They initiated the conversational agent to start the dialogue and afterward left the laboratory so that the participants could talk to the agent privately. Participants opened the laboratory door and called for the experimenter after finishing the dialogue, and once more, they filled in the paper-form STAI and then continued to fill in the rest of the digital questionnaire. After they had finished this questionnaire, the participants were reminded of the second session of the experiment and left the laboratory. Completing this first session took approximately 50 minutes, of which an average of 10.4 minutes accounted for the conversation with the virtual agent.

#### Session 2

Session 2 of the experiment took place exactly 1 week after session 1. The procedure was similar to that of the first session. After participants arrived at the laboratory, they were seated in front of the experiment laptop. They filled in 2 paper-form questionnaires: STAI and WHOQOL-BREF. Next, they started to fill in the digital questionnaire for session 2. After the participants had filled in the CCS, the digital questionnaire instructed them via written instructions to call for the experimenter. They were then able to reread the explanations on CBT, automatic negative thoughts, and the system operation. If no questions remained, the experimenter instructed them to put on the headset and started the conversation dialogue, before leaving the laboratory. Again, participants opened the door and called for the experimenter after finishing the conversation. Lastly, they filled in the paper-form STAI and the remaining digital questionnaire. After that, the experiment was finished. Completing the second session of the experiment took approximately 35 minutes.

### Statistical Analysis

All statistical analyses were carried out using SPSS Version 28.0.1 (IBM Corp). Owing to multiple tests being performed using the present data set, Bonferroni correction was applied in light of the below 5 hypotheses. Consequently, for the statistical analyses regarding the 5 hypotheses, only *P* values of ≤.008 were considered significant.

#### Hypothesis 1

To test for normal distribution of K6 and STAI state anxiety data, the Kolmogorov-Smirnov test was used. Concerning STAI state anxiety, the test indicated a normal distribution of the data (all *P*=.20). To test whether state anxiety was positively influenced or reduced via the experiment, *t*-tests for paired samples were carried out using the mean values of STAI state anxiety data at 4 points of measurement throughout the experiment. For the K6 scores, the Kolmogorov-Smirnov test indicated that the data were not normally distributed (all *P*<.01). Consequently, a Wilcoxon signed-rank test was used to test whether K6 scores differed from each other during the 4 points of measurement.

#### Hypothesis 2

To analyze if changes in psychological distress and anxiety correlated positively with the motivation to change negative thinking, correlations between the SOCRATES, K6 change (K6 at the end of the session – K6 at the beginning of the session), and STAI state anxiety change (STAI at the end of the session – STAI at the beginning of the session) were calculated. A correlational analysis was chosen as motivation had not been accounted for in the previous experiment.

#### Hypothesis 3

To test whether participants’ motivation increased during the experiment, *t*-tests for paired samples were performed after confirming normal distribution of the 3 SOCRATES subscales (Kolmogorov-Smirnov tests: all *P*>.06) to compare the mean scores of the 3 SOCRATES subscales at 3 points of measurement.

#### Hypothesis 4

In order to analyze if cognitive change was positively correlated with the motivation to change negative thinking, correlations of mean CCI and CCS values with the 3 SOCRATES subscales were calculated.

#### Hypothesis 5

Lastly, to analyze if participants who had prior knowledge of automatic negative thoughts or CBT showed larger changes in psychological distress, anxiety, and cognition, a multiple linear regression analysis was performed using prior knowledge of automatic negative thoughts and prior knowledge of CBT (asked in a yes/no format) as predictors and mean changes in K6, STAI state anxiety, CCI, and CCS as dependent variables.

## Results

### General Analysis

The mean values of all questionnaires employed in this experiment are presented in Table 1.

The J-WAI-SR was filled out by participants at the end of both session 1 and session 2. The mean results and significant changes are presented in Table 2, with higher scores indicating a stronger expression of the scales.

Changes in the overall quality of life of participants were investigated by comparing the WHOQOL-BREF total score in session 1 to that in session 2 of the experiment by means of a Wilcoxon signed-rank test. The changes were not significant (Z=–0.89; *P*=.37).

To check for further influences on the experiment’s outcome measures, correlations of the TIPI and GSES with SOCRATES, CCI, CCS, K6, and STAI state anxiety were calculated. Correlations of CCI or CCS with TIPI or GSES scores were not significant (all *P*>.08; Multimedia Appendix 3). However, there were significant correlations with K6, STAI, and SOCRATES scores (Multimedia Appendix 4).

**Table 1:** Mean values of all questionnaires at 4 points of measurement during the experiment.

Questionnaire and topic/subscale		Score range	Score at the beginning of session 1, mean (SD)	Score at the end of session 1, mean (SD)	Score at the beginning of session 2, mean (SD)	Score at the end of session 2, mean (SD)
**K6^a^** [31]						
	Distress	0-24	6.94 (4.79)	4.51 (4.59)	6.14 (4.69)	4.46 (4.66)
**STAI^b^** [29]						
	Trait anxiety	20-80	49.26 (9.98)	—^c^	—	—
	State anxiety	20-80	44.31 (7.17)	37.51 (8.95)	40.29 (8.36)	36.71 (9.42)
**WHOQOL-BREF^d^** [36]						
	Physical health	1-5	3.40 (0.62)	—	3.40 (0.62)	—
	Psychological health	1-5	3.22 (0.68)	—	3.11 (0.66)	—
	Social relationships	1-5	3.30 (0.82)	—	3.28 (0.85)	—
	Environment	1-5	3.31 (0.53)	—	3.31 (0.53)	—
	General	1-5	3.07 (0.73)	—	3.09 (0.72)	—
**QIDS-SR^e^** [26]						
	Depression	0-27	6.66 (4.47)	—	—	—
**GSES^f^** [38]						
	Self-efficacy	0-16	8.40 (4.02)	—	—	—
**SOCRATES^g^ (modification of** [32]**)**						
	Recognition	7-35	18.89 (6.54)	20.43 (7.00)	—	21.17 (7.39)
	Ambivalence	4-20	11.97 (3.79)	12.49 (4.19)	—	13.09 (3.94)
	Taking steps	8-40	22.43 (6.79)	24.66 (8.11)	—	24.71 (7.27)
	Total	19-95	53.29 (14.73)	57.57 (17.11)	—	58.97 (16.76)
**CCI^h^** [34]						
	Cognitive change immediate	0-30	—	14.60 (5.63)	—	14.40 (6.77)
**CCS^i^** [34]						
	Cognitive change sustained	0-54	—	—	21.66 (10.85)	—
**TIPI^j^** [40]						
	Extraversion	2-14	9.03 (2.75)	—	—	—
	Agreeableness	2-14	10.14 (2.23)	—	—	—
	Conscientiousness	2-14	6.17 (2.35)	—	—	—
	Emotional stability	2-14	7.83 (2.53)	—	—	—
	Openness to experience	2-14	9.14 (3.11)	—	—	—
**J-WAI-SR^k^** [42]						
	Goal	4-20	—	12.51 (4.33)	—	13.91 (3.82)
	Task	4-20	—	13.54 (4.02)	—	13.51 (3.23)
	Bond	4-20	—	12.71 (3.73)	—	14.11 (4.11)
	Total	12-60	—	38.77 (11.00)	—	41.54 (9.98)

^a^K6: Kessler Psychological Distress Scale 6-item format.

^b^STAI: State-Trait Anxiety Inventory.

^c^Not applicable.

^d^WHOQOL-BREF: World Health Organization Quality of Life BREF questionnaire.

^e^QIDS-SR: Quick Inventory of Depressive Symptomatology self-report version.

^f^GSES: General Self-Efficacy Scale.

^g^SOCRATES: Stages of Change Readiness and Treatment Eagerness Scale.

^h^CCI: Cognitive Change Immediate scale.

^i^CCS: Cognitive Change Sustained scale.

^j^TIPI: Ten-Item Personality Questionnaire.

^k^J-WAI-SR: Japanese version of the Working Alliance Inventory-Short Revised.

**Table 2:** Japanese version of the Working Alliance Inventory-Short Revised (J-WAI-SR) scores at the end of sessions 1 and 2.

J-WAI-SR^a^ subscale	Score at the end of session 1, mean (SD)	Score at the end of session 2, mean (SD)	*P* value^b^
Goal	12.51 (4.33)	13.91 (3.82)	.02
Task	13.54 (4.02)	13.51 (3.23)	.92
Bond	12.71 (3.73)	14.11 (9.98)	.03

^a^J-WAI-SR: Japanese version of the Working Alliance Inventory-Short Revised.

^b^*P* values are based on a Wilcoxon signed-rank test.

### Reductions of Distress and Anxiety

On average, participants reported low K6 distress (range 0-24; higher scores indicate stronger distress) and moderate STAI state anxiety (range 20-80; higher scores indicate stronger anxiety). Mean values at all 4 points of measurement in the experiment are presented in Table 3.

The significant results of the Wilcoxon signed-rank test for K6 values are presented in Table 4. All other comparisons were not significant (all *P*>.17). Significant *t*-test results of STAI state anxiety are presented in Table 5. The comparison of mean scores at the end of session 1 and the end of session 2 was not significant (*P*=.29). Here, a positive difference in means signifies a reduction of STAI state anxiety scores from the first to the second point of measurement.

**Table 3:** Kessler Psychological Distress Scale 6-item format (K6; distress) and State-Trait Anxiety Inventory (STAI; state anxiety) scores throughout the experiment.

Questionnaire	Score at the beginning of session 1, mean (SD)	Score at the end of session 1, mean (SD)	Score at the beginning of session 2, mean (SD)	Score at the end of session 2, mean (SD)
K6^a^ (distress)	6.94 (4.79)	4.51 (4.59)	6.14 (4.69)	4.46 (4.66)
STAI^b^ (state anxiety)	44.31 (7.17)	37.51 (8.95)	40.29 (8.36)	36.71 (9.42)

^a^K6: Kessler Psychological Distress Scale 6-item format.

^b^STAI: State-Trait Anxiety Inventory.

**Table 4:** Significant results of the Wilcoxon signed-rank test using mean Kessler Psychological Distress Scale 6-item format (K6) scores.

Comparison of means	*Z*	*P*	*r*
K6^a^ at the beginning of session 1 and at the end of session 1	–4.21	<.001	–0.71
K6 at the beginning of session 2 and at the end of session 2	–3.41	<.001	–0.58

^a^K6: Kessler Psychological Distress Scale 6-item format.

**Table 5:** Significant results of t-tests for paired samples using mean State-Trait Anxiety Inventory (STAI) state anxiety scores.

Comparison of means	Difference (mean 1 – mean 2)	*t* test (*df*)	*P* value	*d*
STAI^a^ at the beginning of session 1 and at the end of session 1	6.80	7.71 (34)	<.001	0.97
STAI at the beginning of session 2 and at the end of session 2	3.57	3.05 (34)	.002	0.52
STAI at the beginning of session 1 and at the beginning of session 2	4.03	2.86 (34)	.004	0.48

^a^STAI: State-Trait Anxiety Inventory.

### Correlations of Motivation With Distress and Anxiety

Mean scores for the 3 SOCRATES subscales recognition (range 7-35), ambivalence (range 4-20), and taking steps (range 8-40) are presented in Table 6. Higher scores indicate stronger expression of the respective scale.

In both sessions, there were no significant correlations of K6 and STAI state anxiety changes with the SOCRATES subscales (all *P*>.04; Table 7).

**Table 6:** Stages of Change Readiness and Treatment Eagerness Scale (SOCRATES) scores at 3 points during the experiment.

SOCRATES^a^ subscale	Score at the beginning of session 1, mean (SD)	Score at the end of session 1, mean (SD)	Score at the end of session 2, mean (SD)
Recognition	18.89 (6.54)	20.43 (7.00)	21.17 (7.39)
Ambivalence	11.97 (3.79)	12.49 (4.19)	13.09 (3.94)
Taking steps	22.43 (6.79)	24.66 (8.11)	24.71 (7.27)

^a^SOCRATES: Stages of Change Readiness and Treatment Eagerness Scale.

**Table 7:** Correlations of the Stages of Change Readiness and Treatment Eagerness Scale (SOCRATES) subscales and changes in Kessler Psychological Distress Scale 6-item format (K6) and State-Trait Anxiety Inventory (STAI) state anxiety scores in both experimental sessions.

Variable	Changes in K6^a^ scores in session 1		Changes in K6 scores in session 2		Changes in STAI^b^ state anxiety scores in session 1		Changes in STAI state anxiety scores in session 2	
	Coefficient	*P* value	Coefficient	*P* value	Coefficient	*P* value	Coefficient	*P* value
Recognition subscale of SOCRATES^c^; beginning of session 1	–0.20	.25	—^d^	—	–0.11	.51	—	—
Ambivalence subscale of SOCRATES; beginning of session 1	–0.12	.50	—	—	–0.11	.52	—	—
Taking steps subscale of SOCRATES; beginning of session 1	–0.22	.20	—	—	–0.06	.75	—	—
Recognition subscale of SOCRATES; end of session 1	–0.17	.34	—	—	–0.28	.10	—	—
Ambivalence subscale of SOCRATES; end of session 1	–0.10	.55	—	—	–0.24	.16	—	—
Taking steps subscale of SOCRATES; end of session 1	–0.29	.10	—	—	–0.32	.06	—	—
Recognition subscale of SOCRATES; end of session 2	—	—	–0.30	.08	—	—	–0.30	.08
Ambivalence subscale of SOCRATES; end of session 2	—	—	–0.31	.07	—	—	–0.34	.05
Taking steps subscale of SOCRATES; end of session 2	—	—	–0.33	.05	—	—	–0.36	.04

^a^K6: Kessler Psychological Distress Scale 6-item format.

^b^STAI: State-Trait Anxiety Inventory.

^c^SOCRATES: Stages of Change Readiness and Treatment Eagerness Scale.

^d^Not applicable.

### Increase in Motivation

Results of *t*-test comparisons of the SOCRATES subscales at 3 points of measurement are presented in Table 8. A negative difference in means signifies an increase in the recognition or taking steps subscale from the beginning to the end of an experimental session.

**Table 8:** Results of paired t-test comparisons of the mean values of the 3 subscales of the Stages of Change Readiness and Treatment Eagerness Scale (SOCRATES).

Comparison of means	Difference (mean 1 – mean 2)	*t* (*df*)	*P* value	*d*
Recognition subscale at the beginning of session 1 and at the end of session 1	–1.54	–2.86 (34)	.004	–0.48
Ambivalence subscale at the beginning of session 1 and at the end of session 1	–0.51	–1.55 (34)	.07	–0.26
Taking steps subscale at the beginning of session 1 and at the end of session 1	–2.23	–2.87 (34)	.003	–0.49
Recognition subscale at the end of session 1 and at the end of session 2	–0.74	–1.28 (34)	.11	–0.22
Ambivalence subscale at the end of session 1 and at the end of session 2	–0.60	–1.47 (34)	.08	–0.25
Taking steps subscale at the end of session 1 and at the end of session 2	–0.06	–0.11 (34)	.46	–0.02

### Correlations of Motivation and Cognitive Change Measures

Correlations of CCI and CCS with the 3 SOCRATES subscales are presented in Table 9.

**Table 9:** Correlations of mean Cognitive Change Immediate scale (CCI) and Cognitive Change Sustained scale (CCS) scores with subscales of the Stages of Change Readiness and Treatment Eagerness Scale (SOCRATES).

SOCRATES^a^ subscale	CCI^b^ (same session)			CCS^c^	
	Coefficient	*P* value	Coefficient		*P* value
Recognition subscale at the beginning of session 1	0.29	.09	0.10		.56
Ambivalence subscale at the beginning of session 1	0.30	.08	0.21		.24
Taking steps subscale at the beginning of session 1	0.35	.04	0.45		.006
Recognition subscale at the end of session 1	0.31	.08	0.12		.49
Ambivalence subscale at the end of session 1	0.27	.12	0.21		.23
Taking steps subscale at the end of session 1	0.43	.01	0.48		.004
Recognition subscale at the end of session 2	0.27	.11	0.15		.39
Ambivalence subscale at the end of session 2	0.33	.053	0.12		.50
Taking steps subscale at the end of session 2	0.61	<.001	0.52		.001

^a^SOCRATES: Stages of Change Readiness and Treatment Eagerness Scale.

^b^CCI: Cognitive Change Immediate scale.

^c^CCS: Cognitive Change Sustained scale.

### Effects of Prior Knowledge on Changes in Distress and Anxiety

In the multiple linear regression analysis, prior knowledge of CBT did not emerge as a significant predictor (*F*_2,32_=4.24; *P*=.02).

## Discussion

### General Discussion

The aim of this study’s experiment was to extend the findings of reduced anxiety and distress through CBT carried out using a virtual agent as described by the authors in a past experiment [22]. For this experiment, the following changes were made to the procedure of the past experiment: the CBT scenario script used by the virtual agent was modified and a second CBT session after 1 week was added. Furthermore, to investigate the role of motivation in this specific digital CBT intervention, measures of motivation were added to the experimental procedure. By talking about situations that worried participants and weighing in on how realistic their worries were from alternative points of view, it was aimed to balance potential automatic negative thoughts. In both sessions of the experiment, participants reported experiencing moderate cognitive changes (both CCI and CCS). They stated having noticed changes in their way of thinking during the sessions, which is the aim when using CBT and specifically cognitive restructuring to work on automatic negative thoughts [5,6].

Participants in this study reported mild psychological distress based on the average K6 score. Kessler et al [30] suggested that scores of ≥13 indicate the potential presence of psychological illnesses, which the scores of participants in this study did not indicate. STAI state anxiety scores indicated moderate anxiety at the beginning of both experimental sessions, which could successfully be reduced to low anxiety at the end of both sessions. The results show that CBT with a virtual agent, using a dialogue scenario on the topic of automatic negative thoughts, reduces existing mild distress and anxiety, which is consistent with the findings of Shidara et al [21,22], further confirming the effectiveness of the virtual agent used in these studies. Similarly, Prochaska et al [14] reported that their conversational agent significantly reduced depressive and anxious symptomatology after treatment. There appears to be general success in the use of conversational agents for reducing depressive and anxious symptomatology, as this has been found in various studies employing conversational agents (see [11,12] for reviews). Considering distress, this study’s participants on average reported low distress at all 4 measurement points of the experiment. This distress was higher at the beginning of a session than at the end, indicating that the CBT conversation managed to ease participants’ distress when confronted with their automatic thoughts. These reductions within the 2 sessions were significant changes. While distress was reduced within each session, distress in session 2 did not differ significantly from that previously reported in session 1. Even though the CBT experiment successfully reduced momentary distress, it did not carry over to the following session and ease stress from the very beginning. This might be a result of the average distress being rather low, causing reductions from one session to another to also be rather small. Another possible explanation might be that, in session 2, participants were prompted to focus on and talk about a different automatic negative thought than in session 1. This was a topic they had not discussed with the virtual agent before and therefore might have been related to more distress for the participants. Nonetheless, even when the conversation topic was changed to a new automatic thought, a significant reduction in distress arose. Based on these results, CBT is effective in easing distress when talking about different automatic negative thoughts, even without a human interlocutor. While distress was successfully reduced during the experiment, no changes in participants’ general life satisfaction (as measured via WHOQOL-BREF [35]) were apparent within the 2-week period of the study. A moderate level of satisfaction in all 4 subscales as well as general satisfaction remained constant for the period of the experiment.

Identical to distress, anxiety was significantly reduced from the beginning to the end of both experimental sessions as also seen in the previous study conducted by Shidara et al [22]. At the beginning of both sessions, our participants reported moderate anxiety. After completing the CBT session, this anxiety was reported to be low, which is consistent with the findings of prior studies on conversational agents (see [11,12] for reviews) [13]. Additionally, while participants were moderately anxious at the beginning of both sessions, they were significantly less anxious before session 2 than session 1. We interpret this finding as participants getting used to the experiment and consequently being less anxious at the beginning of the second session. This is a beneficial result for CBT, as generally, it is not desirable for a participant or client to constantly feel anxious before sessions. Therefore, participants getting used to and potentially feeling more comfortable at the prospect of talking about their automatic negative thoughts with a nonhuman agent can be considered a positive finding. This finding is particularly important as CBT using a virtual agent (or communication with a virtual agent in general) is not a common experience for the majority of people.

An improvement in the reception of the virtual agent was also apparent from the J-WAI-SR scores. In human-to-human psychotherapy, therapeutic alliance has been highlighted as a mediating factor of therapy outcomes (see [43] for an analysis). The importance of working alliance between users and conversational agents has been highlighted in the same manner [13,14,44]. The results of this experiment show moderate values comparable to those of other conversational agents [45]. For the subscales “goal” and “bond,” participants reported a significant improvement in session 2 compared to session 1. Specifically, higher scores indicate that participants more strongly felt they and the virtual agent were working toward the same goal and that they felt a stronger connection to the agent. Even though they interacted with a nonhuman interlocutor, their feelings of, for example, being respected and liked by the virtual agent increased. This is a promising result, hinting that CBT with a virtual agent is effective in improving the relationship between the agent and participant and creating an understanding of working toward a goal together. A review by He et al [11] highlighted the role of empathetic responses of conversational agents in creating a bond between them and users and laying the groundwork for a beneficial working alliance. While the presently used script had already been modified to validate participants’ utterances, further enhancements of empathetic responses might increase the perceived bond and working alliance in the context of the present experiment. Future research should further investigate the possibility of building a beneficial therapeutic relationship with a virtual agent, especially since improvements in the alliance of users and virtual agents could result in better intervention outcomes.

Focusing on the results related to motivation, the intervention described in this paper also increased the recognition of negative thinking and the intention (or motivation) to change negative thinking. While participants’ SOCRATES scores of all 3 subscales would be interpreted as low or very low based on existing norms for SOCRATES (it needs to be noted that these norms are based on clients who are in treatment for alcohol problems), participants subjectively reported being more aware of when they thought negatively and reported that they were more actively taking steps to change their negative thinking so that they benefit from it. In this study, changes in distress were not correlated with the motivation to change negative thinking. The reductions in anxiety were in part correlated with motivation, but no clear conclusions could be drawn from these results. Therefore, more research is necessary to gain better insights into this topic. However, cognitive change was significantly positively correlated with the intention to change negative thinking (SOCRATES taking steps subscale), meaning cognitive change was greater for participants who were more motivated to change their negative thinking. This is in line with past research showing that higher motivation leads to better outcomes in psychotherapy [15,17,18] and highlights the importance of ensuring and promoting motivation in a digital intervention to change automatic negative thoughts. The increases in motivation in this study were achieved without employing interventions specifically designed to achieve this effect, such as motivational interviewing [46]. This technique has been used in psychotherapy interventions using conversational agents and has produced satisfactory results [47,48]. Olafsson et al [48] successfully increased their participants’ motivation to change their health-related behaviors by applying this technique and adding humorous expressions to their conversational agent’s dialogue. It would be promising to investigate the changes in outcomes of our experiment when, additionally, techniques to explicitly enhance motivation are incorporated. Furthermore, it needs to be highlighted that due to this part of the analysis being correlational, no causality can be drawn from the results. As there was a significant correlation between motivation and cognitive change, further experimental investigations are crucial to identify the directionality of this relationship. Lastly, while this result was not significant, this study showed a trend of prior knowledge in CBT leading to bigger reductions in distress, but only in session 1 of the experiment. This result could hint at the fact that in CBT for automatic negative thoughts, psychoeducation plays an important role in achieving beneficial outcomes. It might be that because all participants received explanations on CBT and experienced part of CBT themselves with the virtual agent in session 1, they had a similar level of knowledge and no significant influences emerged in session 2. In past research, psychoeducation for depression, anxiety, and distress was highlighted as a measure to help with mood disorders [49], which led to reduced symptomatology. No significant influences of prior knowledge of automatic negative thoughts were found in this study; however, only 5 participants had this prior knowledge that might have been too little to lead to significant effects. A more in-depth investigation of this specific aspect is required in future research.

Going beyond the specific hypotheses postulated in this study, influences of participants’ personality (using the TIPI [40]) and self-efficacy (using the GSES [38]) were taken into account and explored. While some emerging correlations between the scores in these questionnaires and other outcome measures seemed nonsystematic, some interesting patterns showed up within the data. Most prominently, higher scores in the TIPI subscales conscientiousness and emotional stability and in the GSES were linked to lower scores in K6 and STAI state anxiety. Furthermore, they were also linked to lower scores in the SOCRATES subscales recognition and ambivalence. As no hypotheses were postulated concerning these variables prior to the experiment, no further interpretations of these correlations are included in this paper as they would be rather speculative. Nevertheless, the need for personality-adaptive conversational agents has been emphasized in the past (see [50]), and our data further highlight that personality and self-efficacy need to be carefully considered when designing virtual agents for CBT. It appears beneficial to conduct further research on how various personality traits and self-efficacy influence interactions in the context of CBT with a virtual agent.

### Limitations

There are some limitations in the experiment in this study. First, the data consisted of a rather small sample size and did not employ a control group design. To test whether the outcomes produced in this study are caused by the CBT intervention with the virtual agent or simply the interaction itself, an attention-placebo control might be employed, which has been used in past research on psychotherapy interventions [51,52]. Since this analysis showed that existing knowledge of CBT was linked to the outcomes of the CBT experiment, it might also be worth it to include an information control group that only provides participants with information on CBT and automatic negative thoughts without interaction with a virtual agent. Furthermore, the experiment only employed 2 experimental sessions, whereas CBT typically consists of upwards of 10 sessions [23]. It is still unclear how the results obtained in this study might develop with each consecutive additional CBT session. Consequently, no conclusions can be drawn regarding whether the outcomes measured in this study can be generalized to CBT with a virtual agent covering more than two sessions. In future research, these aspects should be worked on and improved to achieve a realistic experimental replication of CBT and more extensive insights into the processes underlying CBT with a virtual agent.

Second, no information about the effects of the experiment on participants’ daily life was asked in this experiment. While the WHOQOL-BREF was employed directly after the CBT experiment to measure changes in participants’ satisfaction with different general aspects of their lives, no checkup after the experiment was conducted to investigate any long-lasting effects after 1 week. Furthermore, participants did not partake in any follow-ups to assess whether the experiment produced lasting effects on their negative thinking. Ideally, by partaking in CBT, the participants should gain long-term effects regarding their way of thinking, leaving it to be more balanced even after the CBT interventions. This should be included in future research as the main goal of CBT (and psychotherapy in general) is to not only produce desired effects in the CBT session but also extend the effects to the daily life of participants.

Lastly, the virtual agent employed in the experiment itself showed some limitations. After the experiment, some participants mentioned that the agent uses a predefined dialog scenario, which makes it unable to change its utterances based on the participants’ responses. While the virtual agent is partially able to adapt its questions based on user utterances, there are still instances where it cannot respond correctly. If, for example, participants asked the virtual agent a question, the agent could not respond to them, but instead continued with the dialogue script. This lack of conversational flexibility could have led to the conversation feeling somewhat unnatural and the participants having negative experiences. Some participants also reported that due to the constraints of voice recognition, the agent cut them off during speech or did not continue with the conversation after they had already stopped talking, potentially impeding a natural conversation flow. Apart from the utterances of the agent, its movement and facial expressions were still quite restricted. To best mimic humans and ensure interactions are as natural as possible, the virtual agent needs to be further improved in its behaviors and flexibility within conversations.

### Conclusions

The experiment in this study showed that interaction with a nonhuman agent can reduce distress and anxiety, which supports the idea that virtual psychotherapy interventions could be used as support in light of not enough human therapists being available in many countries. Furthermore, it reinforced the past findings that higher motivation leads to better outcomes in CBT. This highlights the importance of controlling participants’ motivation to achieve optimal psychotherapy outcomes. Lastly, through interaction with a nonhuman agent, motivation could be increased in our experiment, further highlighting the possibility of CBT with a virtual agent supporting cognitive change even when human psychotherapists are not readily available. If and how these results can be extended to other psychological issues should be the subjects of future research.

## Appendix

Multimedia Appendix 1 Original and modified version of the Stages of Change Readiness and Treatment Eagerness Scale (SOCRATES) questionnaire.

Multimedia Appendix 2 Translation of modified Japanese cognitive behavioral therapy scenarios for sessions 1 and 2 of the experiment.

Multimedia Appendix 3 *P* values of correlations of TIPI and GSES with CCI and CCS.

Multimedia Appendix 4 Correlations of TIPI scales and GSES with different outcome measures.

## Abbreviations

CBT: cognitive behavioral therapy
CCI: Cognitive Change Immediate scale
CCS: Cognitive Change Sustained scale
GSES: General Self-Efficacy Scale
J-WAI-SR: Japanese version of the Working Alliance Inventory-Short Revised
K6: Kessler Psychological Distress Scale 6-item format
QIDS-SR: Quick Inventory of Depressive Symptomatology self-report version
SOCRATES: Stages of Change Readiness and Treatment Eagerness Scale
STAI: State-Trait Anxiety Inventory
TIPI: Ten-Item Personality Questionnaire
WHOQOL-BREF: World Health Organization Quality of Life Questionnaire BREF

## Data Availability

All data generated in this study are available from the corresponding author upon reasonable request.
