# Supplementary material for: Examining the Effects of Cognitive Behavioral Therapy With a Virtual Agent on User Motivation and Improvement in Psychological Distress and Anxiety: Two-Session Experimental Study
Source: JMIR Form Res. 2024 Oct 15;8:e55234. doi: 10.2196/55234 (PMC11522660; doi:10.2196/55234)
Supplement: Multimedia Appendix 1 [file formative_v8i1e55234_app1.docx]

**Multimedia Appendix 1.** Original and modified version of the Stages of Change Readiness and Treatment Eagerness Scale (SOCRATES) questionnaire.

| **Original** | **Modified** |
| --- | --- |
| 1 I really want to make changes in my drinking. | 1 I really want to make changes to my negative thinking. |
| 2 Sometimes I wonder if I am an alcoholic | 2 Sometimes I wonder if I think negatively. |
| 3 If I don't change my drinking soon, my problems are going to get worse. | 3 If I don’t change my negative thinking soon, my problems are going to get worse. |
| 4 I have already started making some changes in my drinking. | 4 I have already started making some changes in my negative thinking. |
| 5 I was drinking too much at one time, but I've managed to change my drinking | 5 In the past, I used to have a lot of negative thoughts, but I’ve managed to change those thoughts. |
| 6 Sometimes I wonder if my drinking is hurting other people. | 6 Sometimes I wonder if my negative thinking is hurting other people |
| 7 I am a problem drinker. | 7 I have a problem with negative thinking. |
| 8 I'm not just thinking about changing my drinking, I'm already doing something about it. | 8 I’m not just thinking about changing my negative thinking, I’m already doing something about it. |
| 9 I have already changed my drinking, and I am looking for ways to keep from slipping back to my old pattern. | 9 I have already stopped thinking negatively, and I'm looking for a way to not go back to my old negative thoughts. |
| 10 I have serious problems with drinking. | 10 I have a serious problem with negative thinking. |
| 11 Sometimes I wonder if I am in control of my drinking. | 11 Sometimes I wonder if I can control my negative thoughts. |
| 12 My drinking is causing a lot of harm. | 12 My negative thinking is causing a lot of harm. |
| 13 I am actively doing things now to cut down or stop drinking. | 13 I am now actively working to reduce or avoid having negative thoughts. |
| 14 I want help to keep from going back to the drinking problems that I had before. | 14 I want help so that I don't go back to my old negative thinking problems. |
| 15 I know that I have a drinking problem. | 15 I know I have a problem with negative thinking. |
| 16 There are times when I wonder if I drink too much. | 16 There are times when I wonder if I have too many negative thoughts. |
| 17 I am an alcoholic. | 17 I can’t stop thinking negatively. |
| 18 I am working hard to change my drinking. | 18 I am working hard to change my negative thoughts. |
| 19 I have made some changes in my drinking, and I want some help to keep from going back to the way I used to drink. | 19 I have made some changes in my way of thinking negatively, and I want help to keep me from going back to my old way of thinking. |
