# Supplementary material for: Examining the Effects of Cognitive Behavioral Therapy With a Virtual Agent on User Motivation and Improvement in Psychological Distress and Anxiety: Two-Session Experimental Study
Source: JMIR Form Res. 2024 Oct 15;8:e55234. doi: 10.2196/55234 (PMC11522660; doi:10.2196/55234)
Supplement: Multimedia Appendix 2 [file formative_v8i1e55234_app2.docx]

**Multimedia Appendix 2.** Translation of modified Japanese cognitive behavioral therapy scenarios for sessions 1 and 2 of the experiment.

Scenario Session One

| **System Utterance Number** | **Utterance** |
| --- | --- |
| 1 | Hello, I am your therapist, Rei. Please tell me your name. |
| 2 | Thank you for coming here to talk to me today. From now on, let’s train with me to face your worries. There are no right or wrong answers. Are you having any troubles right now? Is there anything that bothers you or is difficult to face recently? If you don’t mind, please tell me what you think is hard. |
| 3 | At that time, how did that make you feel? |
| 4 | If 0 is the least painful, and 100 is the worst, how strong was that feeling? |
| 5 | What thoughts came to your mind at that time? |
| 6 | I see. Did you have any other thoughts? If you said everything you can think of, just say „That’s all“. |
| 7 | Please tell me the strongest thought that came to mind. |
| 8 | In situations like this, do you always think this way? |
| 9 | "Did you imagine what might happen in the future as a result of this situation? For example, 'I must be fired from my job in the future as a result from this'.” |
| 10 | What did you think about yourself during that time? |
| 11 | I see. What did you think of other people when you were in this situation? |
| 12 | What are your thoughts about other people and the environment around you? |
| 13 | So how do you think other people would feel about you in that situation? |
| 14 | What do you think you will face in the future? |
| 15 | I see. That’s what you think. The thoughts that come to mind when faced with such an event are called automatic thoughts. You have learned about this kind of thoughts before our conversation. Do you understand? |
| 16 | I would like to know more about the automatic thoughts you mentioned. If your automatic thought is correct, what is the reason for it? |
| 17 | I see. So, what could be a counterargument to your automatic thought? |
| 18 | Can you think of any other way of looking at that situation that you find distressing, other than your current automatic thoughts? |
| 19 | What is the worst possible outcome? |
| 20 | If the worst possible outcome actually happened, how would you deal with it? |
| 21 | And how would you feel, if this outcome happened? |
| 22 | What is the best possible outcome of the situation you are worried about? |
| 23 | I see. So what is the most realistic outcome that can happen going forward? |
| 24 | What effect does believing in your current automatic thoughts have on you? |
| 25 | What effect would modifying your current automatic thoughts have on you? Would you think or behave differently? |
| 26 | What can you do about your automatic thoughts? |
| 27 | Have you tried to change your automatic thoughts before? |
| 28 | Specifically, what is difficult to solve in the situation you described as painful? |
| 29 | What can you do to overcome a difficult situation? |
| 30 | Have you ever been in a similar situation and didn’t get depressed? If so, what can we learn from that experience? |
| 31 | What is the least you can do under this situation, thinking constructively? |
| 32 | What have you done so far to resolve this situation? |
| 33 | What would you do if the same thing happened again? |
| 34 | Can you get help from others? If so, from whom and what kind of support can you get? |
| 35 | What would your family and best friends say if they found out about your automatic thoughts? |
| 36 | I see. If a close friend faced the same situation as you, what advice would you give them? |
| 37 | Are there strengths within you that you are overlooking? How might that help in this situation? |
| 38 | Are you blaming yourself for things out of your control? If you were to be lenient with your responsibilities, what facts would come to mind? |
| 39 | In addition to the automatic thoughts you mentioned, you may find other thoughts as well. If you have an idea, connect it to another thought with a „but“. |
| 40 | I see. How strong is the feeling you were describing at the beginning? Once again, please represent it as a number from 0 to 100. |
| 41 | If you’ve changed your mind, it’s a sign that you have managed to reorganise your thoughts. That’s all for today. Good job for today. Please call me anytime. (s) |

Scenario Session Two

| **System Utterance Number** | **Utterance** |
| --- | --- |
| 1 | Hello, it is good to see you again. How have you been doing? |
| 2 | Last week, we talked about your worries, automatic thoughts and how to question them. Do you remember? |
| 3 | Very good. Our worries and automatic thoughts can be hard to change in the long term. It is important to actively engage with our way of thinking regularly in order to discover automatic thoughts. So today, I want to talk to you about this topic again. Is that okay? |
| 4 | Apart from the difficult situation you have mentioned last week, has there been anything else that has troubled you recently? If you need to, take a moment to think about it. There are no right or wrong answers. |
| 5 | I see. At that time, how did that make you feel? |
| 6 | If 0 is the least painful, and 100 is the worst, how strong was that feeling? |
| 7 | What thoughts came to your mind at that time? |
| 8 | I see. Did you have any other thoughts? If you said everything you can think of, just say „That’s all“. |
| 9 | Please tell me the strongest thought that came to mind. |
| 10 | In situations like this, do you always think this way? |
| 11 | "Did you imagine what might happen in the future as a result of this situation? For example, 'I must be fired from my job in the future as a result from this'.” |
| 12 | What did you think about yourself during that time? |
| 13 | I see. What did you think of other people when you were in this situation? |
| 14 | What are your thoughts about other people and the environment around you? |
| 15 | So how do you think other people would feel about you in that situation? |
| 16 | What do you think you will face in the future? |
| 17 | I see. We have already discussed that certain thoughts that come up in situations like this are called automatic thoughts, right? Let’s talk more about the automatic thoughts you have mentioned today. If your automatic thought is correct, what is the reason for it? |
| 18 | I see. So, what could be a counterargument to your automatic thought? |
| 19 | Can you think of any other way of looking at that situation that you find distressing, other than your current automatic thoughts? |
| 20 | Considering the arguments supporting and counteracting your automatic thoughts, do you think your thoughts are realistic? |
| 21 | I see. What would be the worst possible outcome of the situation you have described? |
| 22 | If the worst possible outcome actually happened, how would you deal with it? |
| 23 | And how would you feel, if this outcome happened? |
| 24 | What is the best possible outcome of the situation you are worried about? |
| 25 | I see. So what is the most realistic outcome that can happen going forward? |
| 26 | What effect does believing in your current automatic thoughts have on you? |
| 27 | What effect would modifying your current automatic thoughts have on you? Would you think or behave differently? |
| 28 | What would your family and best friends say if they found out about your automatic thoughts? |
| 29 | I see. If a close friend faced the same situation as you, what advice would you give them? |
| 30 | What do you think you can do about these specific automatic thoughts? |
| 31 | Have you tried to change these automatic thoughts before? |
| 32 | Specifically, what is difficult to solve in the situation you described as painful? |
| 33 | What can you do to overcome a difficult situation? |
| 34 | Have you ever been in a similar situation and didn’t get depressed? If so, what can we learn from that experience? |
| 35 | What is the least you can do under this situation, thinking constructively? |
| 36 | What have you done so far to resolve this situation? |
| 37 | What would you do if the same thing happened again? |
| 38 | In this situation, is there anyone you can get help from? If so, from whom and what kind of support can you get? |
| 39 | Are there strengths within you that you are overlooking? How might that help in this situation? |
| 40 | Do you think you are being too harsh with yourself in this situation? And maybe blaming yourself for something you do not have control over? |
| 41 | If you were to be lenient with your responsibilities, what facts would come to mind? |
| 42 | In addition to the automatic thoughts you mentioned, you may find other thoughts as well. If you have an idea, connect it to another thought with a „but“. |
| 43 | I see. How strong is the feeling you were describing at the beginning? Once again, please represent it as a number from 0 to 100. |
| 44 | You did very well again, today. Thank you for coming here to discuss your thoughts with me. Call me anytime if you need. (s) |
