# Supplementary figures and images for: Examining the Effects of Cognitive Behavioral Therapy With a Virtual Agent on User Motivation and Improvement in Psychological Distress and Anxiety: Two-Session Experimental Study
Source: JMIR Form Res. 2024 Oct 15;8:e55234. doi: 10.2196/55234 (PMC11522660; doi:10.2196/55234)

**Multimedia Appendix 3.** P-values of correlations of TIPI and GSES with CCI and CCS.


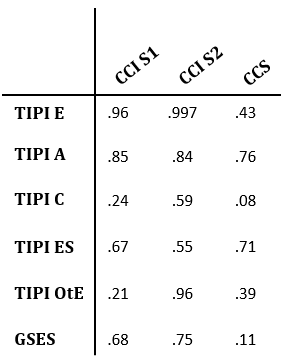

Supplement: Multimedia Appendix 3 [file formative_v8i1e55234_app3.docx]
