# Supplementary material for: Examining the Effects of Cognitive Behavioral Therapy With a Virtual Agent on User Motivation and Improvement in Psychological Distress and Anxiety: Two-Session Experimental Study
Source: JMIR Form Res. 2024 Oct 15;8:e55234. doi: 10.2196/55234 (PMC11522660; doi:10.2196/55234)
Supplement: Multimedia Appendix 4 [file formative_v8i1e55234_app4.docx]

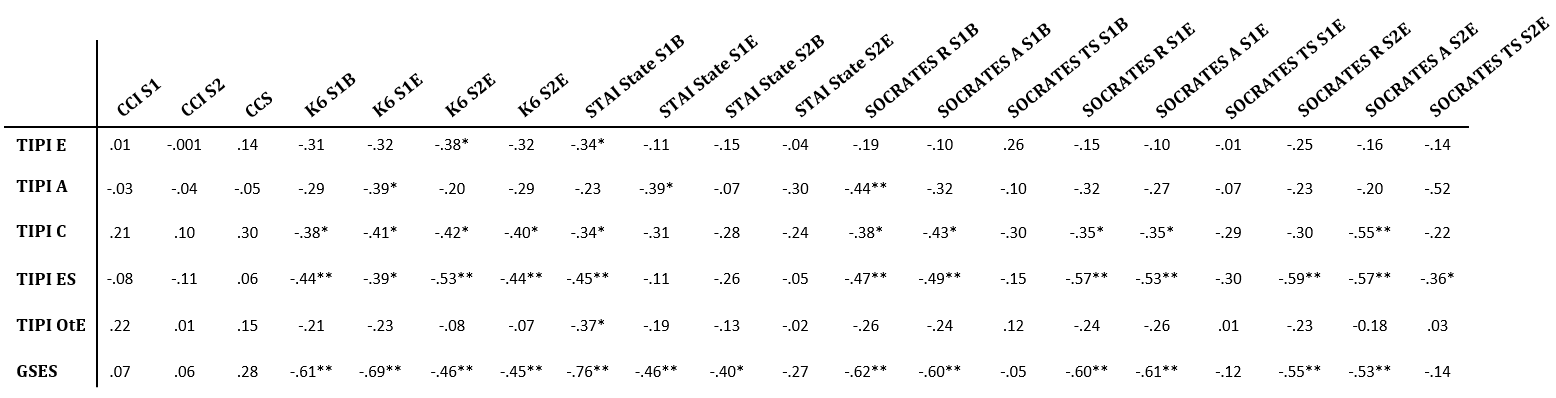
**Multimedia Appendix 4.** Correlations of TIPI scales and GSES with different outcome measures.

*Note.* * *P* < .05, ** *P* < .01.

TIPI E: Extraversion, TIPI A: Agreeableness, TIPI C: Conscientiousness, TIPI ES: Emotional Stability, TIPI OtE: Openness to Experience
